# Guidelines for Evaluation and Management of Cognitive Disorders in HIV-Positive Individuals

**DOI:** 10.1007/s11904-016-0324-x

**Published:** 2016-06-29

**Authors:** Jonathan Underwood, Alan Winston

**Affiliations:** 1 Division of Infectious Diseases, Imperial College London, London, UK; 2 Clinical Trials Centre, Winston Churchill Wing, St Mary’s Hospital, London, W2 1NY UK

**Keywords:** HIV-associated cognitive disorder, Cognitive impairment, HIV, Guidelines

## Abstract

Antiretroviral therapy has revolutionised the treatment for people living with HIV (PLWH). Where antiretroviral coverage is high, the treatment paradigm for HIV-disease is now one of managing the long-term consequences of the virus and its treatment rather than the consequences of untreated HIV-disease such as immunosuppression and opportunistic infections. One such long-term consequence is HIV-associated cognitive impairment which is reported to occur in up to 50 % of treated PLWH and has been associated with poorer outcomes. Given the ageing cohort and increased frequency of comorbidities, the prevalence of symptomatic cognitive impairment may increase with time. High quality evidence for management strategies including screening, diagnosis and treatment of HIV-associated cognitive impairment are lacking and in general guidelines are based on best clinical practice. In this article, we assessed recent guidelines concerning the management of HIV-associated cognitive impairment by performing a systematic review of the MEDLINE database using PubMed. We report that, in general, guidelines from around the world regarding the management of HIV-associated cognitive impairment are converging. Screening is generally not recommended in asymptomatic PLWH. Diagnosis of HIV-associated cognitive impairment should be made only after a comprehensive assessment and exclusion of other potential causes. Antiretroviral therapy forms the cornerstone of management of HIV-associated cognitive impairment and should be guided by plasma and cerebrospinal fluid (CSF) genotype(s).

## Introduction

With the widespread availability of highly tolerable and efficacious antiretroviral therapy (ART) HIV-infection is now a chronic manageable disease. Life expectancy approaches normal for people living with HIV (PLWH) if successfully treated and retained in care [1]. As such, the cohort of PLWH is ageing and the treatment paradigm has shifted from the management of immunosuppression and opportunistic infections to the long-term consequences of HIV-infection and its treatment. HIV-associated cognitive impairment (CI) has been recognised since the early days of the epidemic with approximately 15 % of those with AIDS reported to have co-existing HIV-associated dementia (HAD). Since the introduction of ART, the prevalence of HAD has decreased markedly but there remains a large burden of milder forms of CI affecting up to 50 % of PLWH [2, 3]. It is unclear at the moment whether HIV-infection leads to accelerated or premature ageing [4], but, given that HIV-positive cohorts are ageing, the number of PLWH with symptomatic CI is likely to rise.

**Fig. 1:**
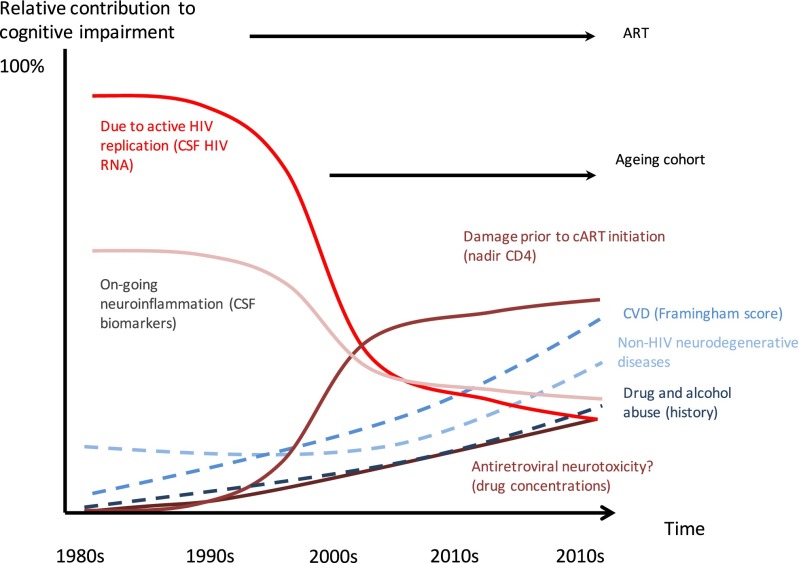
The hypothetical changing aetiology of cognitive impairment in those with HIV (and how it may be possible to assess their contribution in clinical studies)

Research to date has concentrated on the diagnosis and management of HIV-associated CI. However, given the shifting demographics of HIV-positive cohorts, the incidence of other forms of neurodegenerative disease in HIV-positive individuals, also presenting as CI, is likely to increase. This presents a diagnostic conundrum when faced with an HIV-positive patient who has CI. In the pre-ART era, most HIV-positive individuals were young and free of cardiovascular and other end-organ comorbidities. As such, an HIV-positive individual presenting with CI had a high pre-test probability that this condition was HIV-related and the management was clear—namely initiation of ART. In 2016, this is no longer the case. PLWH have an increased burden of comorbidities even when compared to a matched HIV-negative control population. Reported comorbidities includes cardiovascular disease, renal disease, hepatic disease and bone disease which may contribute to or be associated with CI [5]. Given this and the increasing age of the cohort, there are many possible causes of CI in an HIV-positive individual presenting with CI, with HIV-infection being only one (see Fig. 1 for an illustration of the changing aetiology of CI in HIV-positive individuals). In fact, as the majority of PLWH on ART have durable suppression of HIV-replication in both the plasma and central nervous system (CNS) compartments [6•, 7], the likelihood that CI is directly caused by HIV-replication and associated neuroinflammation is now *decreasing*. Further complicating the matter is that in many cases the cause of CI is likely to be multifactorial. CNS injury prior to the initiation of ART is likely to lower the threshold for symptomatic CI, by decreasing ‘physiological reserve’, following further insults such as drug and alcohol misuse, vascular disease [8] and possibly antiretroviral neurotoxicity from chronic treatment [9].

Guidelines are informed by research, and where high quality research is not available guidelines may be based on best clinical practice or opinion of the writing committee. At the current time, the majority of HIV treatment guidelines focus on HIV-related conditions. For example, CI guidelines will generally focus on HIV-associated CI with management of other causes of CI in HIV-positive individuals being less clearly defined.

In this article, we performed a systematic review of recent guidelines concerning the management of CI in HIV. In particular, we reviewed the current guidance surrounding screening, diagnosis and treatment of HIV-associated CI.

## Methodology

We performed a systematic review of the MEDLINE database using PubMed. We searched for HIV treatment guidelines using the search terms ‘HIV’, ‘guideline’ and ‘treatment’. This yielded 227 articles published from 2010 onwards (last search 19 Jan 2016). Furthermore, we also searched PubMed using the terms ‘HIV’, ‘guideline’ and as a third term ‘CNS’, ‘cognitive’ or ‘neuro’ yielding 11, 20 and 1 article(s), respectively. Only articles published in English were reviewed. These were then appraised and if appropriate were selected for further in depth assessment. Additionally, websites of national and international organisations and societies that publish HIV-related guidelines, such as the World Health Organisation (WHO), were searched in case they were not indexed on PubMed. Given the nature of this review, i.e. of guidelines rather than data, qualitative rather than quantitative descriptions of our findings are presented.

National and international guidelines reviewed were published by the Australasian Society for HIV Medicine (ASHM) [10], British HIV Association (BHIVA) [11, 12], European AIDS Clinical Society (EACS) [13, 14], French HIV Expert Group [15], the International Advisory Panel on HIV Care (IAPAC) [16], International Antiviral Society [17], Infectious Diseases Society of America (IDSA) [18], Italian HIV Guidelines Group [19, 20], Korean Society for AIDS [21], Quebec HIV Care Committee [22], Thai National HIV Guidelines Working Group [23], United States Department of Health and Human Services (DHHS) [24, 25] and the WHO [26–28]. In addition, important guidelines concerning HIV-associated CI not published by national or international bodies, such as the MIND Exchange Working Group, were also used for reference purposes [29••, 30••, 31].

## Results

### Screening for CI

Screening for CI in both HIV-positive and HIV-negative populations is a controversial topic. In HIV-negative populations, screening is generally not recommended in those without symptoms [32, 33]. This is largely due to the lack of efficacious treatment and concerns about over-diagnosis and increased anxiety in those identified as having mild CI at screening where prognosis is uncertain. Valcour et al provide an excellent review of the issues surrounding screening for CI in PLWH [29••]. Although they do not make a firm recommendation either for or against screening, they make the point that, if performed, screening should use a strategy of testing multiple cognitive domains because of likely sub-cortical nature of HIV-associated CI. This effectively precludes the use of the mini-mental state examination (MMSE) which was originally designed to differentiate between patients with functional and organic dementias and primarily tests ‘cortical’ domains [34].

Most HIV treatment guidelines do not make any specific recommendations about screening for CI. Of the ones that do, there is considerable variation in guidance reflecting the uncertainties in the literature. The EACS v8.0 guidelines published in 2015 recommend screening only symptomatic HIV-positive individuals ‘without highly confounding conditions’ at diagnosis and before ART initiation and then as indicated based on symptoms [14]. This is a subtle change from guidance published a year earlier that recommended screening all PLWH every 2 years regardless of symptoms [13]. The EACS screening method involves asking three questions: ‘Do you experience frequent memory loss?’; ‘Do you feel that you are slower when reasoning, planning activities, or solving problems?’ and ‘Do you have difficulties paying attention?’. Responses of ‘yes, definitely’ to at least one constitutes a positive screening test necessitating further investigation. This is different from guidance from the consensus report of the Mind Exchange Program (2013) who recommend screening within 6 months of diagnosis, before ART initiation, every 6–12 months if high risk, every 12–24 if low risk and immediately if there is any clinical deterioration (grade of evidence 5 [Oxford Centre for Evidence-Based Medicine, CEBM [35]) and grade of recommendation D) [30••]. In contrast to the EACS guidelines that specify asking the ‘three questions’, the Mind Exchange Working Group recommend using a screening tool appropriate to the goal in mind (i.e. identification of HAD or milder forms of CI), clinical environment, clinician expertise and availability. The tests they prefer are similar to those recommended by Valcour et al. [29••] with the international HIV dementia scale (iHDS) noted in both for its speed and ease of use. The Italian ART guidelines (2011) recommend screening all PLWH with the EACS ‘three questions’ (see above). Additional screening methods they mention were the iHDS or the MMSE although it was not clear the situation where one would be preferred over the others. The BHIVA routine monitoring guidelines (2011) are more circumspect about screening and mention a ‘general assessment of mood and cognitive function’ should be performed pre-ART initiation and at least annually with history and clinical examination as appropriate [12]. In those with symptoms suggestive of cognitive decline, investigations are recommended for HIV-related CI as well as excluding possible alternative causes. This is similar to guidance published by the IDSA in their primary care HIV guidelines [18]. The WHO recommend that routine screening for mental health disorders should be provided for key populations of PLWH in order to optimise health outcomes and improve ART adherence [27]. However, the preferred screening method and frequency are not specified.

### Diagnosis of CI

In general, there is agreement between guideline bodies regarding the diagnosis of HIV-associated CI. A comprehensive assessment including a thorough history and examination, screening for depression, neuropsychological testing, cerebral MRI scanning and lumbar puncture is recommended by all guidelines that have specific sections regarding HIV-associated CI [10, 14, 19, 30••]. This is helpfully presented in the form of an algorithm by the Italian HIV Guidelines Group and EACS [14, 19]. To further guide management plasma and cerebrospinal fluid (CSF) HIV RNA and genotyping is recommended, if available and appropriate. This is primarily to identify the small proportion of patients with symptomatic CI who have discordant suppression of HIV-replication in plasma and CSF compartments—so called ‘CSF escape’ [7]. Different thresholds of discordance have been proposed with EACS defining escape as detection of HIV RNA in CSF, despite undetectable HIV RNA in plasma, or a CSF HIV RNA 1 log_10_ copies/mL higher than concomitant plasma level (if detectable) [14]. The additional purpose of CSF examination is to identify other causes of CI in appropriate situations (e.g. neurosyphilis). With an ageing population, examination of CSF biomarkers to distinguish between Alzheimer’s, HIV-associated and other dementias may become more important. However, at present, quantification of specific CSF biomarkers other than HIV RNA is not recommended. The ASHM guidelines (2009) also recommend cerebral MR spectroscopy, if available, to aid diagnosis but this is not specifically recommended in other guidelines.

Although there is no clear consensus on the exact tests that should be used as part of the neuropsychological assessment, all guidelines recommend a comprehensive battery, testing several cognitive domains and reference the international expert consensus guidelines commonly known as the ‘Frascati criteria’ which in the supplementary information recommend several preferred tests for each cognitive domain [31]. The Mind Exchange Working Group make the useful point that the tests selected should be validated in the language and culture of the population tested with appropriate normative data available to interpret the results [30••].

### Management of HIV-associated CI

Antiretroviral management of PLWH is a rapidly progressing field. The recent results of the INSIGHT-START trial [36••] have provided clarity about when to start ART at higher CD4+ cell counts. This has simplified ART treatment somewhat, with all recent treatment guidelines advocating ART at any CD4+ cell count. However, in recent years, all guidelines previously recommended initiation of ART in those diagnosed with HIV-associated CI regardless of CD4+ lymphocyte count so it is questionable whether this new data has impacted management in those presenting with CI who are not receiving ART (BHIVA level of evidence 1C; Grading of Recommendations Assessment, Development and Evaluation [GRADE] [37]). Earlier treatment may lead to a reduced incidence of HIV-associated CI [38], but preliminary results from the INSIGHT-START neurological sub-study suggest there to be no specific cognitive benefit in those initiating ART immediately with a CD4+ lymphocyte count above 500 cells/μL versus waiting to initiate ART before the CD4+ lymphocyte count falls to around 350 cells/μL [39].

Only the previous ASHM guidelines recommend the use of specific antiretrovirals in patients diagnosed with HIV-associated CI with suspected favourable CNS pharmacokinetics, such as zidovudine (AZT) [10]. The BHIVA guidelines recommend standard ART aside from efavirenz (GRADE evidence 1C) and nucleoside sparing regimens, such as protease inhibitor (PI) monotherapy, which should be avoided [11]. They also make the specific point that the clinical penetration effectiveness (CPE) score, which provides a simplified scoring system based on each antiretroviral’s likely CNS exposure [40], should not influence therapeutic decisions in patients with HIV-associated CI commencing ART. The DHHS guidelines also recommend the avoidance of efavirenz in those with HAD, favouring darunavir- or dolutegravir-based regimens [25]. The MIND Exchange Working Group is similarly guarded regarding the use of CPE score to guide choice of ART, acknowledging the uncertainty in the literature [30••].

**Fig. 2:**
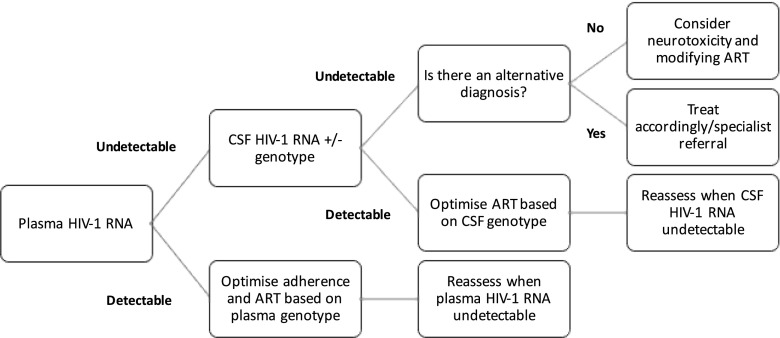
Proposed algorithm for the management of confirmed cognitive impairment (assuming no confounding condition) in HIV-positive individuals already receiving antiretroviral therapy

In those receiving ART, the management of HIV-associated CI is more complicated. Again, an algorithmic approach is generally advocated depending on various factors such as CSF HIV RNA and availability of CSF HIV genotyping and low-copy CSF HIV RNA assays [30••]. It is generally recommended that ART should be optimised based on plasma and CSF genotypes with consideration given to likely antiretroviral CNS exposure [10, 11, 14, 19, 24, 30••]. The general approach to a HIV-positive patient with confirmed CI with no confounding condition is summarised in Fig. 2. Although CSF genotyping is not available in all healthcare settings, this recommendation to assess for the presence of HIV drug resistance mutations in CSF samples in guidelines allows healthcare providers to advocate funding for this laboratory test from healthcare funding agencies.

### Monitoring/Follow-up

In common with many disease areas, data about the practicalities of clinical management, such as how frequently to review patients and in what setting, are lacking. This is due to numerous factors such as the heterogeneity of provision of clinical care across the globe, physician availability and patient expectation. HIV-associated CI is no different, and guidance is based largely on longitudinal studies and expert opinion. There is agreement between the various bodies in that a sufficient duration (i.e. weeks) should elapse after an intervention before testing is repeated due to the likely kinetics of CNS HIV-replication and potential recovery. The ASHM recommend repeat lumbar puncture, neuropsychological testing and MRI after 12 weeks of a new antiretroviral regimen [10]. For those with HAD, neuropsychological testing is recommended every 6 months with a repeat lumbar puncture reserved for those with evidence of relapse. EACS recommend repeating CSF examination and other tests after at least 4 weeks [14]. The Italian HIV Guidelines Group recommend re-evaluation after 3–12 months using neuropsychological testing depending on the severity of CI with tests repeated annually in those with the mildest disease or who have recovered [19]. The MIND Exchange Working Group recommend more frequent monitoring [30••]. For those with HIV-associated CI not receiving ART, reassessment is recommended monthly if possible. Those diagnosed with HAD or ‘mild neurocognitive disorder’ (MND) commencing ART should be monitored clinically, initially every 3 months and then every 6 months until a plateau in response is seen and then annually. In those with asymptomatic impairment, monitoring is recommended initially after 6 months and then annually thereafter.

## Conclusions

Guidelines from around the world on the management of HIV-associated CI are converging. In general, screening for CI is not recommended in HIV-positive populations without symptomatology and diagnosis of HIV-associated CI should be made only after a comprehensive assessment and exclusion of other potential causes. ART and adherence forms the cornerstone of management of HIV-associated CI, and any modifications to ART in subjects with HIV-associated CI should be guided by both plasma and CSF genotype(s) in the case where HIV-viraemia is detected.
